# Editorial: Hiding features in myeloid cells: metabolism preference in different disease models

**DOI:** 10.3389/fimmu.2025.1565258

**Published:** 2025-02-11

**Authors:** Jie Shen, DuoYao Cao

**Affiliations:** ^1^ Department of Gastrointestinal (GI) Surgery, Tongji Hospital, Tongji Medical College, Huazhong University of Science and Technology, Wuhan, China; ^2^ Department of Biomedical Sciences, Cedars-Sinai Medical Center, Los Angeles, CA, United States

**Keywords:** myeloid cells, metabolism, glycolysis, TCA cycle, lipid metabolism, protein metabolism

Myeloid cells form the cornerstone of the human immune defense, playing pivotal roles in innate immunity, tissue repair, and inflammation. Acting as sentinels, these cells detect and eliminate potential threats in a healthy immune system. Historically, myeloid cell functions were thought to be primarily driven by cytokine and chemokine stimulation. However, emerging research has unveiled a critical role for metabolic shifts in regulating myeloid cell function across various disease contexts.

In a healthy immune system, myeloid cells adeptly utilize glucose, fatty acids, and amino acids to generate energy, ensuring efficient responses to threats. However, metabolic dysfunction can compromise these cells, leading to energy deficits and an accumulation of glucose, lipids, and proteins. This metabolic imbalance often results in apoptosis and necrosis at inflammation sites, increasing susceptibility to infections, tumors, and chronic diseases such as atherosclerosis, diabetes, obesity, and Alzheimer’s disease (1–7).

This Research Topic ‘Hiding Features in Myeloid Cells: Metabolism Preference in Different Disease Models’ has investigated the interplay between metabolism and myeloid cell function, providing valuable insights into their roles in different diseases and potential therapy strategy:

**Cancer Therapy and Myeloid Metabolism**: Tumor-associated macrophages (TAMs) and myeloid-derived suppressor cells (MDSCs) rewire their metabolic programs to favor tumor growth, immune evasion, and therapy resistance. Enhanced fatty acid oxidation (FAO), cholesterol metabolism, and glycolysis in these cells create a tumor-supportive microenvironment. Clinically, metabolic reprogramming can explain the limited efficacy of current immunotherapies in tumors with high TAM or MDSC infiltration. Drugs targeting lipid metabolism, such as inhibitors of CD36 or fatty acid transport proteins, have shown potential in preclinical models by reducing TAM-driven immune suppression and boosting T-cell-mediated anti-tumor responses. Combining metabolic inhibitors with immune checkpoint blockade or chemotherapy could significantly improve outcomes in cancers like glioblastoma and melanoma​. (Essakhi et al.; Chen et al.; Goldmann et al.).

**Neurodegenerative Diseases**: Metabolic dysfunction in microglia, driven by lipid overload and sTREM2 dysregulation, has been implicated in diseases like Alzheimer’s. Elevated sTREM2 levels in cerebrospinal fluid (CSF) correlate with microglial activation during the early stages of neurodegeneration, suggesting its potential as both a biomarker and a therapeutic target. Strategies to modulate microglial metabolism—such as reducing lipid accumulation or enhancing phagocytic efficiency—could slow disease progression. Clinical trials investigating sTREM2 agonists or lipid metabolism modulators may hold promise for altering the trajectory of Alzheimer’s and other neurodegenerative conditions (Lin et al.).

**Hematopoietic Cell Metabolism in Regenerative Medicine**: Hematopoietic stem cells (HSCs) exhibit distinct metabolic profiles that support their self-renewal and differentiation capacities. High oxidative phosphorylation and fatty acid oxidation activity in HSCs underlie their resilience and therapeutic potential in bone marrow transplantation and regenerative medicine. Clinically, modulating these metabolic pathways could improve the engraftment and functionality of HSCs in treating hematological disorders (Zhang et al.).

**Cardiovascular Diseases and Metabolic Adaptations**: In atherosclerosis, lipid-laden macrophages contribute to plaque instability and chronic inflammation. Targeting metabolic pathways, such as inhibiting CD36-mediated lipid uptake, has shown potential in stabilizing plaques and reducing macrophage-driven inflammation. Emerging therapeutic approaches aim to reprogram these foam cells toward a less inflammatory phenotype, thereby improving cardiovascular outcomes and reducing the risk of acute events like myocardial infarction (Chen et al.).

**Immunometabolism in Chronic Inflammation and Autoimmunity**: Aberrant heme metabolism in myeloid cells, driven by dysregulated heme oxygenase-1 (HO-1) activity, exacerbates inflammatory conditions such as rheumatoid arthritis and inflammatory bowel disease. Pharmacological modulation of HO-1 activity could restore immune balance by reducing oxidative stress and inflammation. Clinically, HO-1 inducers or inhibitors may emerge as adjunct therapies for managing autoimmunity and chronic inflammatory disorders (Consonni et al.).

**Combination Therapies and Personalized Medicine**: The integration of metabolic reprogramming strategies with existing therapies offers opportunities for personalized medicine. For instance: 1) Combining lipid metabolism inhibitors with immunotherapy could enhance the efficacy of checkpoint inhibitors in resistant tumors. 2) Using sTREM2-targeted therapies in conjunction with amyloid-beta clearance strategies might amplify therapeutic effects in Alzheimer’s patients. 3) Modulating metabolic pathways in myeloid cells during hematopoietic cell transplantation could improve patient outcomes in leukemia and lymphoma​.

Metabolic interventions represent a promising frontier in precision medicine. By targeting specific metabolic vulnerabilities in myeloid cells, we can not only reshape the immune landscape but also enhance the efficacy of current therapies across multiple diseases. Moving forward, clinical trials should focus on validating these metabolic strategies in diverse patient populations, with particular attention to biomarkers like sTREM2 and CD36 for patient stratification and treatment monitoring. The transformative potential of these insights lies in their ability to bridge basic research and clinical practice, offering hope for tackling some of the most challenging diseases of our time—cancer, neurodegeneration, and metabolic disorders—through the lens of immunometabolism.
